# Remote Ischemic Conditioning Improves Attention Network Function and Blood Oxygen Levels in Unacclimatized Adults Exposed to High Altitude

**DOI:** 10.14336/AD.2019.0605

**Published:** 2020-07-23

**Authors:** Sijie Li, Cong Han, Karam Asmaro, Shanyi Quan, Ming Li, Changhong Ren, Jun Zhang, Wenbo Zhao, Jiali Xu, Zhiwen Liu, Peng Zhang, Lingling Zhu, Yuchuan Ding, Kai Wang, Xunming Ji, Lian Duan

**Affiliations:** ^1^Beijing Key Laboratory of Hypoxic Conditioning Translational Medicine, Xuanwu Hospital, Capital Medical University, Beijing, China.; ^2^Department of Neurosurgery, Fifth Medical Center of PLA General Hospital, Beijing, China.; ^3^Department of Neurosurgery, Henry Ford Health System, Detroit, MI, USA.; ^4^Department of Health, Xizang Military Region of PLA, Xizang, China.; ^5^Laboratory of Neuropsychology, Department of Neurology, The First Affiliated Hospital of Anhui Medical University, Anhui Province, China.; ^6^Institute of Military Cognition and Brain Science, Academy of Military Medical Sciences, Beijing, China.; ^7^Department of Neurosurgery, Wayne State University School of Medicine, Detroit, MI, USA.

**Keywords:** remote ischemic conditioning, high altitude, hypoxia, attentional network function

## Abstract

Remote ischemic conditioning (RIC) confers protection on major organs from hypoxic/ischemic injuries; however, its impacts on attention network function and blood oxygen levels in unacclimatized adults exposed to high altitudes have yet to be elucidated. In this study, we recruited 120 healthy male volunteers, of which one was exposed to high altitude and the other was exposed to low altitude. The two cohorts were further divided into RIC and sham control groups. The attentional network test (ANT) was performed to evaluate cognitive function before and after RIC treatment. Other outcomes such as heart rate, blood pressure, blood oxygen saturation, cerebral tissue oxygenation index (CTOI), and cerebrovascular hemodynamic indices were also evaluated. Prior to RIC treatment, there were no significant differences in orienting or executive function between the treatment and control arms of either cohort. Alerting function was significantly lower in the high-altitude cohort than in the low-altitude cohort. There were significant reductions in both blood oxygen and CTOI in the high-altitude cohort relative to the low-altitude cohort, while the pulse index (PI) of the former cohort was significantly increased. After RIC treatment, there was a significant difference in alerting function between the high-altitude RIC group and its associated control. The CTOI of the treatment group increased from 60.39±3.40% to 62.78±4.40%, and blood oxygenation also improved. Furthermore, this group showed a significant reduction in its PI. Exposure to high-altitude environments had a significant impact on alerting function, blood oxygen, CTOI, and PI. RIC ameliorated changes in attentional function, as well as blood oxygen and CTOI, suggesting that it potentially alters cerebrovascular compliance upon exposure to high altitude.

High altitude has been shown to cause a series of pathophysiological changes via hypoxia, low atmospheric pressure, cold, and other environmental factors [1], which may lead to acute, subacute, or chronic mountain sickness depending on the duration of exposure [2, 3]. On plateaus, the reduced partial pressure of oxygen can compromise the adequate supply of oxygen to the brain tissue, such as the sensitive and hypoxia-prone tissues in the limbic system and hippocampus, and thereby cause cognitive impairment [4, 5]. Previous studies have demonstrated that acute or chronic exposure to hypoxia causes cognitive dysfunction and impairment by affecting working memory [6], perception [7], vision [8], decision making, learning skills [9], and a constellation of other functions; all of these effects are derived from disturbance of neuronal physiology [10, 11]. Therefore, there is great significance in the search for effective interventions to prevent or cure cognitive impairment caused by exposure to high altitude [12].

Remote ischemic conditioning (RIC) is a systemic strategy in which several cycles of ischemia followed by reperfusion in the limbs confer protection on distant vital organs [13, 14]. RIC has been demonstrated to benefit patients with cardiocerebrovascular disease, exerting strong neuroprotective effects [15, 16]. Intriguingly, RIC has been shown to delay the onset of acute mountain sickness in normobaric hypoxia [16], improve oxygen saturation, and decrease hypoxic pulmonary vasoconstriction in high-altitude areas [17], suggesting a potential benefit in the prevention of high-altitude disease [18]. However, it remains unclear whether RIC can improve neuronal hypoxic tolerance during high-altitude exposure and help mitigate cognitive dysfunction.

Recently, some studies have demonstrated that the attentional network can be disturbed by brief exposure to high altitude [19-21]. In addition, scores on the Attention Switching Task (AST) have been demonstrated to decrease with acclimatization (exposure to high altitude for 6 days) [22]. However, these studies used only small samples with different ethnicities and locations. Evidence for an impact of high-altitude exposure on attention remains scarce. In this study, we aim to investigate the impact of high-altitude exposure on attention and cognitive function in young healthy volunteers who are unacclimatized to high-altitude areas and evaluate the effects of RIC on cerebral protection in high-altitude hypoxia.

## MATERIALS AND METHODS

### Study design and subjects

We recruited two cohorts of subjects. The first cohort included 60 young healthy male volunteers who were newcomers to the region and had arrived at an altitude of 3,700 meters on the Tibetan Plateau 3 days before entering the study. The other cohort included 60 young healthy male volunteers who remained at an altitude of 42 meters on the plains and had never lived in a high-altitude region (>3,000 meters). These two cohorts of subjects were randomly assigned to RIC or sham group (n=30 in each group). Other inclusion criteria for this study included the following: (1) age between 18 and 30 years old; (2) right-hand dominance, have normal or corrected-to-normal vision, absence of intellectual disability, and no history of neurological/psychiatric illness or hemorrhagic diseases; (3) no history of intravascular thrombosis in either of the upper limbs.

**Figure 1.**
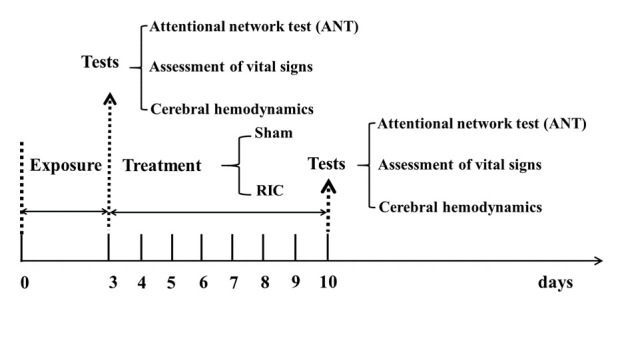
Schematic diagram of RIC treatment and examination.

### Interventions

All subjects underwent RIC or sham-RIC treatment for one week, beginning on the third day of exposure (Fig. 1). The RIC treatment consisted of 5 cycles of bilateral upper limb ischemia for 5 min followed by reperfusion for 5 min, performed twice a day for a total of 7 days. The treatment was carried out using an automatic electric control device (patent number ZL200820123637.X, China). Limb ischemia was induced by inflating blood-pressure cuffs to 200 mmHg. At the beginning of every RIC treatment, the device records the heart rate and blood pressure in real time. In the event of discomfort or lack of tolerance, the patient could abort the RIC process at any time. The high-altitude and low-altitude sham groups underwent the same process, except with a cuff pressure of 60 mmHg.

### Assessment of vital signs

The heart rate and blood pressure of each subject was acquired before and after RIC treatment. We monitored blood oxygen saturation (SpO_2_) with a portable oxygen saturation apparatus designed for hospital wards (Datex-Ohmeda, Madison, WI, USA), and we measured the cerebral tissue oxygen index (CTOI) with a cerebral oxygen monitor (EGOS-600, Jiangsu, China).

### Attentional network test

The attentional network test (ANT), as described by Fan *et al.* in 2002 [23], was administered before and after treatment. All stimuli were displayed on a computer screen. Subjects were required to determine whether a centrally located arrow pointed to the left or the right and were instructed to respond via two buttons. In the cued reaction time conditions, one of four cue types was provided: no cue, a central cue, a double cue, or a spatial cue to alert the participant to the possible location of an array of arrows (the flanker condition) that would subsequently appear on the screen. The arrow could appear above or below the fixation point and with or without flankers. The efficiency of the three attentional networks was assessed by measuring how much response times are influenced by alerting cues, spatial cues, and flankers, as follows: alerting effect = RT_no cue_ - RT_central cue_, where the larger the difference is, the more efficient the alerting network is; orienting effect = RT_central cue_ - RT_spatial cue_, where the larger the difference is, the more efficient the orienting network is; conflict effect = RT_incongruent_ - RT_congruent_, where the larger the difference is, the less efficient the conflicting network is.

### Cerebral hemodynamic assessments

All cerebral hemodynamic data were collected using a transcranial doppler (DWL Doppler-Box, Germany) according to applicable specifications and technical standards before and after one week of treatment. The peak systolic blood flow velocity (PSV), end-diastolic blood flow velocity (EDV), and mean flow velocity (MFV) of the subjects’ bilateral middle cerebral artery (MCA) were recorded, and the pulse index (PI) was calculated.

### Statistical analysis

For continuous data, the mean ± standard deviation (SD) was used to summarize the data; independent-samples t-tests or Mann-Whitney U tests were performed to detect differences between groups. For binary data, frequency or percentage was used to summarize data, and between-group comparisons were performed via the chi-squared test or Fisher’s exact test as appropriate.

All data were analyzed using SPSS 19.0 (IBM Statistics) with a significance level of p<0.05 (two sides).

**Table 1:** Demographic data.

Characteristics	High altitude		Low altitude		P
	Sham (A) (n=30)	RIC (B) (n=30)	Sham (C) (n=30)	RIC (D) (n=30)	
**Age (years)**	19.70±1.42	19.17±1.53	19.07±1.82	19.50±1.50	0.819
**Height (cm)**	174.37±4.42	174.93±4.88	176.70±5.91	176.83±4.19	0.212
**Body mass (kg)**	65.43±6.29	63.70±5.32	66.10±5.51	69.46±5.4	0.092
**Education (years)**	9.87±0.97	9.97±1.19	9.87±1.07	9.9±1.39	0.734

Data are presented as the mean ± standard deviation. RIC, remote ischemic conditioning.

## RESULTS

### Demographics

The demographic and clinical characteristics of the subjects in the four groups are summarized in Table 1. No differences were observed in regards to age (high altitude: 19.70±1.42 years in the sham-RIC group, 19.17±1.53 years in the RIC group; low altitude: 19.07±1.82 years in the sham-RIC group, 19.50±1.50 years in the RIC group, P>0.05 each), height (high altitude: 174.37±4.42 cm in the sham-RIC group, 174.93±4.88 cm in the RIC group; low altitude: 176.70±5.91 cm in the sham-RIC group, 176.83±4.19 cm in the RIC group, P>0.05 each), weight (high altitude: 65.43±6.29 kg in the sham-RIC group, 63.70±5.32 kg in the RIC group; low altitude: 66.10±5.51 kg in the sham-RIC group, 69.46±5.4 kg in the RIC group, P>0.05 each), or education (high altitude: 9.87±0.97 years in the sham-RIC group, 9.97±1.19 years in the RIC group; low altitude: 9.87±1.07 years in the sham-RIC group, 9.9±1.39 years in the RIC group, P>0.05 each).

**Table 2:** Vital signs and ANT data before RIC.

Characteristics	High altitude		Low altitude		P (A vs. C)
	Sham (A) (n=30)	RIC (B) (n=30)	Sham (C) (n=30)	RIC (D) (n=30)	
**Heart rate**	81.4±16.9	78.30±11.68	77.43±2.07	76.83±6.75	0.772
**Blood pressure**	116.73±12.46	120.23±14.09	116.56±13.92	119.14±15.41	0.315
	80.20±8.92	77.83±11.58	76.80±10.56	77.46±9.55	0.723
**Blood oxygen**	90.93±2.07	91.13±2.07	94.23±2.53	93.67±2.13	<0.001
**CTOI**	59.13±2.53	58.14±2.79	65.95±3.62	65.15±3.64	<0.001
**ANT data**					
Alerting	28.53±19.62	27.87±12.85	42.70±27.30	43.16±19.74	0.025
Orienting	35.13±19.05	33.63±16.86	42.03±25.56	39.89±27.18	0.138
Executive	117.83±46.27	121.70±32.74	126.13±36.99	122.84±37.97	0.425

Data are presented as the mean ± standard deviation. RIC, remote ischemic conditioning; CTOI, cerebral tissue oxygenation index; ANT, attentional network test.

### Assessment of vital signs and ANT data before RIC

No differences were observed between the high-altitude and low-altitude groups in terms of heart rate or blood pressure. In the high-altitude cohort, the blood oxygen and CTOI of the sham-RIC group were 90.93±2.07 and 59.13±2.53, respectively, and those of the RIC group were 91.13±2.07 and 58.14±2.79; those of the low-altitude cohort were 94.23±2.53 and 65.95±3.62 in the sham-RIC group and 93.67±2.13 and 65.15±3.64 in the RIC group .Both parameters were significantly different between the two cohorts (P<0.001). The alerting function of the high-altitude cohort was 28.53±19.62 and 27.87±12.85 in the sham-RIC group and RIC group, respectively; these values were significantly lower than those of the low-altitude group (42.70±27.30 and 43.16±19.74 in the sham-RIC group and RIC group, respectively, P=0.025, Table 2). In contrast, there were no significant differences in orienting or executive function between the high- and low-altitude groups.

**Table 3:** Vital signs and ANT data from the low-altitude cohort after RIC or sham treatment.

Characteristics	Sham (n=30)	RIC (n=30)	P
**Heart rate**	75.92±5.46	72.45±6.12	0.84
**Blood pressure**	117.25±16.73	110.46±20.14	0.71
	78.82±8.27	76.16±9.73	0.43
**Blood oxygen**	92.39±1.81	91.56±1.42	0.09
**CTOI**	66.13±2.41	67.56±3.02	0.67
**ANT data**			
Alerting	42.43±18.56	40.18±20.04	0.75
Orienting	39.75±25.13	38.11±24.75	0.97
Executive	120.35±36.07	121.45±34.21	0.77

Data are presented as the mean ± standard deviation. RIC, remote ischemic conditioning; CTOI, cerebral tissue oxygenation index; ANT, attentional network test.

### Assessment of vital signs and ANT data from the low-altitude cohort after RIC or sham treatment

There was no significant difference between the low-altitude RIC and low-altitude sham groups in any of the vital sign assessments or ANT results after one week of treatment, leading us to conclude that RIC has no influence on healthy volunteers in low-altitude regions (Table 3).

### Assessment of vital signs and ANT data from the high-altitude cohort after RIC or sham treatment

There was no significant difference between the high-altitude RIC and sham groups in terms of heart rate or blood pressure after one week of treatment. The CTOI of the RIC group was 62.78±4.40%, which was higher than that of the sham-RIC group (60.39±3.40%, P=0.022), and the blood oxygenation was also higher with RIC than with sham treatment (92.87±1.68 vs. 90.53±2.06, P<0.01). Together, these results indicate that blood oxygen saturation and CTOI were significantly improved by RIC treatment. There were no significant differences in orienting (sham-RIC group vs. RIC group: 38.53±18.01 vs. 43.23±25.75, P>0.05) or executive function (sham-RIC group vs. RIC group: 115.47±31.60 vs. 117.53±50.59, P>0.05). However, after RIC or sham treatment of the high-altitude cohort, the alerting score of the sham-RIC group was 26.96±18.18, while that of the RIC group was significantly higher at 48.80±24.32 (P<0.001; Table 4).

**Table 4:** Vital signs and ANT data from the high-altitude cohort after RIC or sham treatment.

Characteristics	Sham (n=30)	RIC (n=30)	P
**Heart rate**	76.04±4.41	78.3±8.81	1.000
**Blood pressure**	116.19±15.31	115.53±13.13	0.159
	72.13±9.07	73.56±10.38	0.148
**Blood oxygen**	90.53±2.06	92.87±1.68	<0.01
**CTOI**	60.39±3.40	62.78±4.40	0.022
**ANT data**			
Alerting	26.96±18.18	48.80±24.32	<0.001
Orienting	38.53±18.01	43.23±25.75	0.091
Executive	115.47±31.60	117.53±50.59	0.417

Data are presented as the mean ± standard deviation. RIC, remote ischemic conditioning; CTOI, cerebral tissue oxygenation index; ANT, attentional network test.

### Cerebrovascular hemodynamic indices in high-altitude conditions

No differences were observed between the high-altitude and low-altitude cohorts in terms of the PSV, EDV or MFV in the bilateral MCA of the volunteers. The PI, however, was significantly (P<0.01) higher bilaterally in the high-altitude group than in the low-altitude group (high-altitude cohort: PI of the left MCA (LMCA) was 0.93±0.09% and 0.92±0.12%, respectively, in the sham-RIC and RIC groups; PI of the right MCA (RMCA) was 0.93±0.11% and 0.93±0.13%, respectively, in those two groups; low-altitude cohort: PI of the LMCA was 0.83±0.10% and 0.82±0.09%, respectively, in the sham-RIC and RIC groups; PI of the RMCA was 0.81±0.13% and 0.80±0.08%, respectively, in those two groups; Table 5). The PSV, EDV, and MFV showed no obvious changes after treatment except for a significant (P<0.05) decrease in the PI bilaterally. The PI of the bilateral MCA in the RIC group (PI of LMCA: 0.86±0.12, PI of RMCA: 0.84±0.08) was lower than that in the sham-RIC group (PI of LMCA: 0.92±0.10, PI of RMCA: 0.90±0.12; P<0.05; Table 6).

**Table 5:** CVHIs of the high-altitude and low-altitude cohorts before RIC.

		High altitude		Low altitude		P
		Sham (A) (n=30)	RIC (B) (n=30)	Sham (C) (n=30)	RIC (D) (n=30)	(A vs. C)
**LMCA**	**PSV (mm/s)**	104.59±19.52	105.16±21.01	107.49±17.82	106.12±21.75	0.552
	**EDV (mm/s)**	43.84±12.74	44.17±11.92	51.21±12.33	51.55±9.84	0.798
	**MFV (mm/s)**	65.28±11.17	65.89±10.45	67.12±12.33	66.26±11.29	0.854
	**PI (%)**	0.93±0.09	0.92±0.12	0.83±0.10	0.82±0.09	<0.01
**RMCA**	**PSV (mm/s)**	104.34±20.34	103.6±19.78	104.24±20.19	105.14±19.51	0.656
	**EDV (mm/s)**	43.22±14.26	42.97±13.41	52.67±13.28	53.19±15.55	0.673
	**MFV (mm/s)**	65.98±11.45	65.29±9.56	63.44±13.67	64.86±10.71	0.908
	**PI (%)**	0.93±0.11	0.93±0.13	0.81±0.13	0.80±0.08	<0.01

Data are presented as the mean ± standard deviation. CVHI, cerebral vascular hemodynamics index; RIC, remote ischemic conditioning; LMCA, left middle cerebral artery; RMCA, right middle cerebral artery; PSV, peak systolic blood flow velocity; EDV, end-diastolic blood flow velocity; MFV, mean flow velocity; PI, pulse index.

## DISCUSSION

In this study, we found that, compared with the low-altitude cohort, the high-altitude cohort had diminished alerting function, while their orienting and executive function remained unchanged. In addition, this reduction in alerting function was significantly improved after one week of RIC treatment in the high-altitude cohort population, suggesting that RIC treatment is a promising strategy to cure cognitive impairment during high-altitude exposure.

The human attention network model proposed by Posner and Petersen [24] postulates that attention includes three distinct functions-alerting, orienting, and executive function. These three functions are subserved by distinct and largely independent neural networks. The attentional component of alerting is defined as the ability to maintain an alert state synchronously with the phasic response to the incoming signal. The orienting network is defined as the ability to select information among numerous incoming sensory signals. Executive function concerns the resolution of conflicts in information. There are few reports focusing on the variation in attentional network function under different environmental conditions.

**Table 6:** CVHIs of the high-altitude and low-altitude cohorts after RIC.

		High altitude		P	Low altitude		P
		Sham (A) (n=30)	RIC (B) (n=30)		Sham (C) (n=30)	RIC (D) (n=30)	
**LMCA**	**PSV (mm/s)**	106.30±20.05	105.91±19.98	0.934	107.78±19.13	106.07±21.82	0.847
	**EDV (mm/s)**	45.64±8.54	48.01±10.00	0.276	50.89±10.56	51.18±9.74	0.094
	**MFV (mm/s)**	65.86±12.04	67.31±12.90	0.617	66.23±10.52	64.88±13.46	0.736
	**PI (%)**	0.92±0.10	0.86±0.12	0.035	0.85±0.08	0.84±0.14	0.858
**RMCA**	**PSV (mm/s)**	105.81±19.84	103.63±19.33	0.632	103.47±20.13	102.96±18.55	0.476
	**EDV (mm/s)**	46.61±10.00	47.82±9.82	0.600	50.71±11.29	49.14±9.87	0.549
	**MFV (mm/s)**	66.35±12.83	66.43±12.78	0.978	64.84±15.13	65.37±13.34	0.724
	**PI (%)**	0.90±0.12	0.84±0.08	0.037	0.81±0.11	0.82±0.16	0.101

Data are presented as the mean ± standard deviation. CVHI, cerebral vascular hemodynamics index; RIC, remote ischemic conditioning; LMCA, left middle cerebral artery; RMCA, right middle cerebral artery; PSV, peak systolic blood flow velocity; EDV, end-diastolic blood flow velocity; MFV, mean flow velocity; PI, pulse index.

Our study showed that blood oxygen saturation was lower in the high-altitude group than in the low-altitude group. According to the ANT, the alerting function of the high-altitude subjects was reduced compared to that of the low-altitude subjects. Alerting is the ability to prepare for a sensory signal, allowing one to achieve and maintain a highly sensitive state of vigilance in which one is ready for any incoming stimuli. Godfrey *et al.* [25] found that fatigue could reduce individual cognitive function. Reduced oxygen saturation plays a pivotal role in the development of central fatigue-that is, fatigue originating within the central nervous system. We hypothesized that unacclimatized subjects, when suddenly exposed with high altitudes, would have difficulty maintaining a vigilant state as a result of central fatigue. However, we were unable to find any discrepancy in orienting or executive function when participants were subjected to a higher altitude in our study, and further investigation might be necessary to corroborate or dispute these findings.

RIC is a phenomenon in which a brief period of ischemia in one organ or tissue protects another organ or tissue against sustained ischemia-reperfusion injury [15, 26]. Previous studies have found that RIC can reduce the recurrence of stroke, attenuate inflammation and improve cerebral circulation in patients with symptomatic intracranial arterial stenosis [15, 16, 27]. RIC has also been shown to be safe and efficacious in cerebral small-vessel disease patients with mild cognitive impairment [28]. Another study has shown that intermittent hypoxia can improve exercise tolerance and central fatigue in severe hypoxic conditions [29]. However, to date, no study has focused on the safety and cerebroprotective effects of RIC at high altitudes. In this study, 30 young male subjects unacclimatized to high-altitude conditions underwent RIC treatment for one week. The treatment was well tolerated without any significant change in heart rate or blood pressure; no serious adverse reactions occurred. We also found that RIC treatment was safe for high-altitude hypoxia intervention and brought some improvement in alerting function. Although the mechanism of cerebroprotection secondary to RIC treatment is not yet clear, we found that RIC increased oxygen saturation, suggesting that improved microcirculation in the brain tissue might contribute to relieving central fatigue and enhancing alerting function.

The current study has some limitations. The study recruited only a small sample size, and the follow-up period was short; these limitations may have introduced some biases influencing the results. At the same time, although this study found improvement in attention network function after RIC treatment, the exact mechanism through which RIC exerts that effect was not investigated in depth. Finally, since only male volunteers were recruited in this study, the consequent sex bias may impact the generalizability of these results.

In conclusion, short-term exposure to high altitudes produced a significant impact on alerting function, and RIC treatment is a useful strategy to mitigate the reduction in alerting function in high-altitude or other hypoxic environments. Further studies are necessary to further confirm these results and to explore the underlying mechanisms.
